# Association of 24‐h movement behaviors during Covid‐19 pandemic with spinal musculoskeletal disorders in undergraduate students: a cross‐sectional study

**DOI:** 10.1002/hsr2.70121

**Published:** 2024-12-18

**Authors:** Gracielle de Jesus Santos, William Rodrigues Tebar, Danilo Rodrigues Pereira da Silva, Eduardo da Silva Alves, Diego Giulliano Destro Christofaro, David Ohara

**Affiliations:** ^1^ Programa de Pós‐Graduação em Ciências da Saúde ‐ PPGCS/UESC Brazil; ^2^ Centro de Pesquisa Clínica e Epidemiológica, Hospital Universitário – USP Brazil; ^3^ Department of Physical Education Federal University of Sergipe São Cristóvão Brazil; ^4^ Faculty of Health Sciences Universidad Autónoma de Chile Providencia Chile; ^5^ University Centre for Rural Health, Uralba St Lismore 2480 New South Wales Australia; ^6^ Programa de Pós‐Graduação em Ciências do Movimento – UNESP Brazil; ^7^ Programa de Pós‐Graduação em Educação Física ‐ PPGEF/UESB‐UESC Brazil; ^8^ Departamento de Saúde I ‐ DSI/UESB Brazil

**Keywords:** Covid‐19, exercise, lifestyle, musculoskeletal pain, sleep, spine

## Abstract

**Background and Aims:**

Musculoskeletal disorders (MSD) are an important health problem, and the Covid‐19 pandemic affected several lifestyle and health aspects worldwide, including moderate‐to‐vigorous physical activity (MVPA), sedentary behavior (SB) and sleep time, which compose the 24‐h movement guidelines (24‐hMG). It is unclear whether meeting 24‐hMG during pandemic have been associated with MSD in adult population and this study aimed to analyze the association of meeting 24‐hMG with spinal MSD in undergraduate students during Covid‐19 pandemic.

**Methods:**

A sample of 71 undergraduate students were assessed (24.0 ± 6.6 years, 52% of women). MVPA was assessed by accelerometer, while daily time of SB and sleep were assessed by questionnaires. Spinal MSD was assessed by the Nordic questionnaire. Logistic regression models analyzed the association of meeting 24‐hMG (individually and combined) with MSD, independently of age, sex, and body mass index.

**Results:**

Spinal MSD affected 78.9% of sample. Only 11.3% of sample fully met the 24‐hMG. Participants who did not meet both MVPA and SB guidelines were more likely to have MSD in low back (OR:3.45, *p* = 0.049) and spine (OR:3.78, *p* = 0.048) even after multiple adjustment. Otherwise, not meeting SB guideline was inversely associated with upper back MSD (OR:0.22, *p* = 0.012).

**Conclusion:**

Meeting 24‐hMG during Covid‐19 pandemic were differently associated with spinal MSD, where combination of MVPA and SB shows to be protective against MSD in low back and in spine. However, the determinants of association between meeting SB guideline and higher upper back MSD still needs to be investigated.

## 1 INTRODUCTION

Musculoskeletal disorders (MSD) are the leading cause of functional disability in the world and a leading cause of severe pain and loss of productivity in adults.^1^ The MSD have been considered as a health and economic problem, which comprise several conditions affecting the skeletal musculature, bones, joints, and connective tissues, being an important cause of loss of function and pain occurrence.^2^ The burden of MSD tends to be higher in countries low‐middle income countries ^3^ and a national health survey observed that about 27 million with 18 years or older reported to have chronic problem in the spine region in Brazil, which represents 18.5% of Brazilian population.^4^

The MSD in the spine region has been associated with different lifestyle behaviors in adult population. A number of previous studies observed that sufficient levels of moderate‐to‐vigorous physical activity (MVPA) was inversely associated with MSD in the spine region.^5^ The adequate sleep duration also has been considered as a protective factor against MSD.^6^ In contrast, sedentary behavior (SB) showed to be positively associated with occurrence of MSD in neck, upper back and low back in adult population.^7^

These three lifestyle behaviors compose the 24‐h movement guidelines,^8^ which have been a novel lifestyle recommendation considering the different contexts of daily life simultaneously. Emerging evidence showed a positive association of meeting 24‐movement guideline with several physical and psychological healthy conditions.^9^ However, there is no evidence about the association of meeting 24‐h movement guidelines with MSD in adult population.

Young adults have a hectic lifestyle, mainly if they were at the undergraduate, reconciling daily activities with curricular ones.^10^ This troubled condition was accentuated in the Covid‐19 pandemic context, with the advent of remote classes due to the social distancing policies to mitigate the widespread of Coronavirus transmission, along with psychosocial distress, fear of virus infection, worries about health of family and friends, and increase in symptoms of anxiety and depression.^11^ In addition, the lifestyle behaviors that compose 24‐h movement guideline also have been substantially affected during pandemic, where was reported a significant reduction in physical activity levels, increase in sedentary behavior, and impairments in sleep time, which have been mutually associated.^12^, ^13^ Nevertheless, Brazilian undergraduate students have reported a high prevalence of MSD in the spine region, reaching 74.9%.^14^

In this sense, it is still not known whether the lifestyle behaviors during Covid‐19 pandemic could be associated with MSD among young adults. Therefore, the aim of present study was to analyze the association of meeting 24‐h movement guidelines during Covid‐19 pandemic with MSD in spine region among undergraduate students.

## 2 METHODS

### 2.1 Sample

This observational study has a cross‐sectional design, being composed of a sample of undergraduate students of both genders, aged 18 years or older, from the State University of Santa Cruz (UESC/BA). The research procedures were conducted in compliance with the Strengthening the Reporting of Observational Studies in Epidemiology guidelines. The participants were invited through instant messaging application groups and social media. As a dissemination strategy, a digital card was created, with explanation of the research purposes, contact of the researchers and a link directing to an online pre‐registration form, to recruitment of those who were interested in participate.

Eligible participants were those who had 18 years or older at enrollment; was a regular student at UESC; and signed the informed consent form for participation. The study did not include those who had restrictions to physical activity, to understand the questionnaires, and with limited internet access that made it impossible to fill out the online questionnaires during Covid‐19 pandemic period. The data were collected between August and December 2021, comprising the delivery and collection of accelerometry devices and answer to an online questionnaire. If the participant was unable to answer the online survey, the questionnaire was administered by a restricted video call interview between researcher and participant.

This study recruited a total of 94 participants. Groups were created on instantaneous message app, to facilitate the communication with participants, to send an illustrative manual with instructions on the correct use of the device and reminders of use to prevent forgetting, in addition to clarifying possible doubts. However, there were exclusions due to incorrect accelerometer use (*n* = 1), not answer the questionnaires (*n* = 4), and for reporting to have Covid‐19 diagnosis (*n* = 18). Participants with Covid‐19 diagnosis were excluded due to the numerous pain symptoms reported by infected patients ^15^ and for being a period that vaccination had not started in Brazil and severe cases of Covid‐19 were more prevalent, which could confound the results. At the end, a sample of 71 undergraduate students were included in data analysis.

### 2.2 Ethical issues

This study is part of the Research Project entitled “Physical activity, sedentary behavior and sleep: 24‐h movement behaviors and health indicators in adults”, proposed by the Department of Health Sciences of the of the State University of Santa Cruz, DCS/UESC, approved by the Ethics Committee on Ethics in Research with Human Beings of the UESC. The research procedures were in accordance with the ethical principles of the Declaration of Helsinki and a written consent form was obtained from the participants before any data collection.

### 2.3 Musculoskeletal disorders

The presence of MSD were investigated by the Brazilian version of Nordic Musculoskeletal Symptoms Questionnaire. This questionnaire contained the sketch of a human figure in posterior position, divided into 9 anatomical regions (neck, shoulders, elbows, wrists/hands, upper back, low back, hip/thighs, knees, and ankles/feet), where the participant answer about the presence of MSD in each region through the period of 12 months. For the purpose of the present study, only the spine region was considered: neck, upper back and low back. A cluster of these three regions were further created, named as “spinal MSD”, considering the presence of MSD in at least one of the three body regions.

### 2.4 Physical activity

The physical activity was objectively assessed by accelerometer device wGT3X‐BT (ActiGraph 11 Corp, Pensacola, FL, USA). Because the data collection was carried out during Covid‐19 pandemic period, the procedures of biosafety were adopted. Before the participants receive the devices at home, they were sanitized with 70% alcohol, with the belts washed with soap and water, according to procedures recommended by Health Surveillance Institutions and by the manufacturer of the equipment, ACTIGRAPH. After drying, the devices were packed into disposable plastic bags, along with the diary of activities and the term of receipt of the equipment. The researchers and participants have only face‐to‐face meeting at the moment of delivery and collection of the equipment, respecting the safety protocols.

The participants were instructed to use the accelerometer positioned at the right side of waist line during all the waking time for 1 week. It was recommended to take off the device only for personal hygiene and water situations (i.e., bathing, swimming, taking rain). Data were considered valid from participants who had wear time equal to or greater than 600 min per day for four or more days, being at least 1 weekend day. To validate the periods of wear time, it was used the automatic wear time algorithm.

For the accelerometry data curation, the results of ActiGraph device are presented through units of acceleration (counts), which were summed over a specific time window (epoch) of 60 s, which provides the counts per minute and allowing to convert into minutes of activity, being a usual metric for the adult population. The cutoff point for MVPA was ≥2690 counts per minute,^16^ being calculated in minutes per week. The guideline for sufficient MVPA from 24‐h movement behaviors considered a weekly amount of 150 or more minutes of MVPA.

### 2.5 Sedentary behavior

The SBQ questionnaire developed by ^17^ was used to assess the sedentary behavior of sample. This instrument quantifies the time spent per day in nine different behaviors: watching television, computer use, sitting listening music, sitting talking to the phone, sitting doing office tasks, sitting reading, sitting playing musical instruments, sitting doing crafts, and sitting driving. These items were score for a typical week day and for a typical weekend day (responses were: i. none; ii. 15 min or less; iii. 30 min; iv. 1 h; v. 2 h; vi. 3 h; 4 h; vii. 5 h; viii. 6 h or more). The sedentary time was converted into mean hours per day and the weighted average was calculated (week day hours*5 + weekend hours*2, divided by 7).

Because the sedentary behavior guideline from the 24‐h movement behaviors considers both total sedentary time and screen‐based leisure time, the time spent watching television and computer use were also quantified separately. Participants who presented less than 8 daily hours of total sedentary time, being less than 3 h of screen‐based leisure time, were considered as meeting sedentary behavior guideline.

### 2.6 Sleep

The sleep time of the sample was calculated by the Brazilian version of PSQI ‐ Pittsburgh Sleep Quality Index questionnaire.^18^ This instrument asks about the time that participant usually go to sleep, how much minutes the participant takes to sleep after going to bed, and what time the participant usually gets up. The sleep time was calculated by the difference between the time that participant go to sleep and the time of gets up, excluding the time that takes to sleep after going to bed, being accounted in hours of sleep. The sleep guideline of 24‐h movement behaviors considers the cutoff point of 7 or more daily sleep hours as sufficient sleep time. For the purpose of present study only consider the sleep time, the other questions of PSQI were not considered.

### 2.7 Covariates

The variables of sex, age, and body mass index were considered as covariates of this study analysis. The self‐reported information about body mass (kg) and height (m) were used to calculate the body mass index (BMI = kg/m²) of the sample, since clinical evaluations were suspended during Covid‐19 quarantine.

### 2.8 Statistical analysis

The descriptive characteristics of sample was presented in median and interquartile range (IQR) for continuous variables and in absolute and relative frequencies for categorical variables. The Man‐Whitney U test was used to median values comparison, while chi‐square test was used to comparison of proportions. Logistic regression models were used to analyze the association of meeting 24‐h movement behavior guidelines with spinal MSD, by considering participants who met guidelines as reference group, against those participants who did not meet guidelines for inference of likelihood to have MSD. Crude (Model 1) and multiple models were created with adjustment for age, sex, and body mass index (Model 2) and addition of meeting the remaining guidelines (Model 3). A posteriori statistical power was calculated according to the formula proposed by Rosner,^19^ considering the absolute difference of MSD prevalence between groups, the sample size of each group, and an alfa error of 5%. All statistical analyses were performed in IBM SPSS software version 25.0 (IBM Corporation, Armonk, NY, USA), considering a statistical significance level of *p* < 0.05 for 2‐sided tests.

## 3 RESULTS

The final sample of 71 undergraduate students presented a median age of 24.0 years (IQR: 21.0–29.0) and was composed of 52% of women. The participants presented a median body mass index of 22.5 kg/m² (IQR: 20.6–26.4), besides of 8.1 (6.3–11.3) median hours per day of sedentary behavior, 86.0 (42.0–158.0) median minutes of MVPA per week, and 6.5 (6.0–8.0) median hours of sleep time per day. When considered the presence of MSD in spine region, participants had lower median MVPA than those who did not have spinal MSD (68.5 vs. 150.0, *p* = 0.002). The descriptive characteristics of sample according to the presence of MSD are presented in Table 1.

**Table 1:** Descriptive characteristics of the sample (*n* = 71).

Variables	Spinal disorders^a^ (*n* = 56)	No spinal disorders (*n* = 15)	*p*value
Age (years), median (IQR)	24.0 (21.0–28.8)	23.0 (21.0–36.0)	0.538
Female gender, n (%)	41 (73.2)	11 (73.3)	>0.99
Body Mass Index (kg/m²), median (IQR)	22.4 (20.5–26.5)	22.6 (20.8–25.8)	0.978
Sedentary behavior (hours/day), median (IQR)	8.1 (6.6–11.3)	8.0 (5.0–9.3)	0.39
MVPA (minutes/week), median (IQR)	68.5 (35.3–145.3)	150.0 (93.0–205.0)	**0.002**
Sleep time (hours/day), median (IQR)	6.8 (6.0–8.0)	6.0 (5.0–7.0)	0.17

*Note*: Bold values were statistically significant at *p* < 0.05 level.

Abbreviations: IQR, Interquartile range; MVPA, Moderate‐to‐vigorous physical activity.

^a^ considering the presence of neck, upper back or low back musculoskeletal disorders.

**Figure 1:**
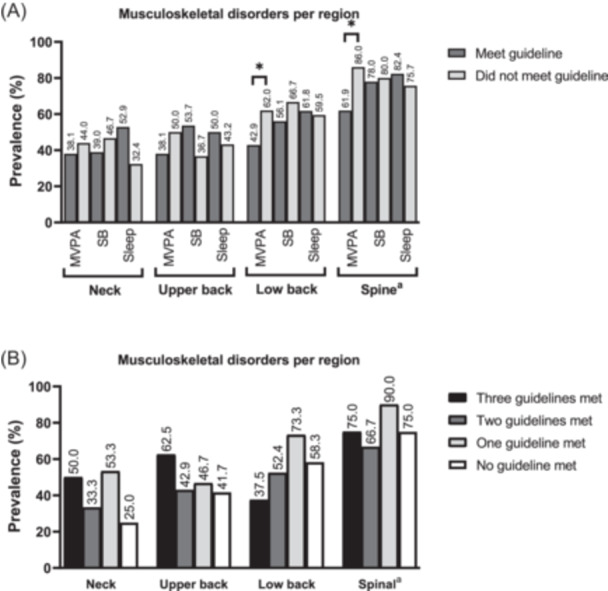
Prevalence of musculoskeletal disorders in undergraduate students during the Covid‐19 pandemic, according to meet 24‐h movement guidelines (*n* = 71). MVPA, Moderate‐to‐vigorous physical activity; SB, Sedentary behavior. a: considering musculoskeletal disorders in neck, upper back or low back. Panel A musculoskeletal disorders according to meet 24‐hour movement guidelines individually. Panel B musculoskeletal disorders according to the clustered meeting of 24‐hour movement guidelines.

It was observed an overall prevalence of spinal MSD of 78.9% in the sample, where 42.3% (*n* = 30) reported MSD in the neck, 46.5% (*n* = 33) in the upper back, and 60.6% (*n* = 43) in the low back region. Considering multiple areas, the prevalence of MSD both at the neck and upper back was 5.6% (*n* = 4), at the neck and low back was 12.7% (*n* = 9), and at the upper and low back was 9.9% (*n* = 7), and at the three spine regions (simultaneously at the neck, upper back and low back) was 21.1% (*n* = 15). Regarding to meet the 24‐h movement behavior guidelines, 11.3% (*n* = 8) of the sample met the three guidelines, 29.6% (*n* = 21) met two from the three guidelines, 42.3% (*n* = 30) met only one from the three guidelines, and 16.9% (*n* = 12) of sample did not meet any guideline. The Figure 1 presents the prevalence of spinal MSD according to specific regions and according to meet the movement guidelines. The Panel A shows that participants who met MVPA guideline showed lower prevalence of MSD in low back (42.9% vs. 62.0%, *p* = 0.048) and in spine (61.9% vs. 86.0%, *p* = 0.02) when compared to those who did not meet MVPA guideline. No difference in the prevalence of MSD was observed according to the number of guidelines met in any specific spinal region – Panel B.

The median values of movement behavior indicators were compared according to spinal MSD in specific regions – Table 2. A lower MVPA level was observed in participants that reported to have MSD, when compared to those that reported not to have MSD. While participants with no spinal MSD presented a median of 150 weekly minutes of MVPA (reference group), those who reported MSD in the neck presented 77.0 median weekly minutes (*p* = 0.01 for U Mann‐Whitney test), in the upper back presented 62.0 median weekly minutes (*p* = 0.006 for U Mann‐Whitney test), and in low back presented 63.0 median weekly minutes of MVPA (*p* = 0.001 for U Mann‐Whitney test). No significant difference was observed in sedentary behavior and sleep time according to MSD.

**Table 2:** Comparison of movement behaviors according to spine musculoskeletal disorders per region in undergraduate students (*n* = 71).

	No spinal MD	Neck MD^a^	Upper back MD^a^	Low back MD^a^
	Median (IQR)	Median (IQR)	Median (IQR)	Median (IQR)
MVPA (minutes/week)	150.0 (93.0–205.0) *Reference group*	**77.0 (35.8–164.0)** ***p*** = **0.012**	**62.0 (28.5–148.0)** ***p*** = **0.006**	**63.0 (35.0–137.0)** ***p*** = **0.001**
Sedentary behavior (hours/day)	8.0 (5.0–9.3) *Reference group*	8.3 (6.2–12.1) *p* = 0.427	9.1 (6.8–11.6) *p* = 0.276	8.1 (6.3–11.5) *p = *0.419
Sleep time (hours/day)	6.0 (5.0–7.0) *Reference group*	7.0 (6.0–8.0) *p* = 0.051	7.0 (6.0 ‐ 8.0) *p* = 0.154	6.5 (6.0 ‐ 8.0) *p* = 0.221

*Note*: Bold value was statistically significant at *p* < 0.05 level.

Abbreviations: MD, Musculoskeletal disorders; MVPA, Moderate‐to‐vigorous physical activity.

^a^ compared against reference group.

The association of meeting movement behavior guidelines with MSD in neck and upper back was analyzed by logistic regression and is presented in Table 3. It was observed that participants who did not meet the sedentary behavior guideline were less likely to have MSD in upper back when compared to those who meet this guideline, even after adjustment for meeting the remained guidelines (Odds ratio: 0.22 [95% confidence interval: 0.07; 0.72], *p* = 0.01), showing a posteriori statistical power of 29%. An inverse association between neck MSD and not meeting sleep guideline was not statistically confirmed in multiple adjusted model (Odds ratio: 0.38 [95% confidence interval: 0.14; 1.03], *p* = 0.06), showing a posteriori statistical power of 41.4%. No association of MSD with specific guidelines combination (MVPA + sleep; MVPA + sedentary behavior; sleep + sedentary behavior) or guidelines clustering (none, one, two or three guidelines met) was observed.

**Table 3:** Association of meeting the 24‐h guideline components during Covid‐19 pandemic with low back and spinal musculoskeletal disorders in undergraduate students (*n* = 71).

	Neck MD			Upper back MD		
	Model 1	Model 2	Model 3	Model 1	Model 2	Model 3
	OR (95%CI)	OR (95%CI)	OR (95%CI)	OR (95%CI)	OR (95%CI)	OR (95%CI)
*Individual guidelines*						
MVPA						
Met guideline	Reference	Reference	Reference	Reference	Reference	Reference
Did not meet	1.28 (0.45; 3.62) *p* = 0.65	1.32 (0.46; 3.82) *p* = 0.60	1.20 (0.40; 3.61) *p* = 0.75	1.63 (0.57; 4.60) *p* = 0.36	1.63 (0.54; 4.91) *p* = 0.38	2.29 (0.68; 7.66) *p* = 0.18
SB						
Met guideline	Reference	Reference	Reference	Reference	Reference	Reference
Did not meet	1.37 (0.53; 3.55) *p* = 0.52	1.67 (0.60; 4.62) *p* = 0.33	1.75 (0.60; 5.15) *p* = 0.31	0.50 (0.19; 1.31) *p* = 0.16	**0.26 (0.09; 0.80) *p*** = **0.02**	**0.22 (0.07; 0.72) *p*** = **0.01**
Sleep						
Met guideline	Reference	Reference	Reference	Reference	Reference	Reference
Did not meet	0.43 (0.16; 1.12) *p* = 0.08	0.39 (0.15; 1.06) *p* = 0.06	0.38 (0.14; 1.03) *p* = 0.06	0.76 (0.30; 1.94) *p* = 0.57	0.87 (0.32; 2.34) *p* = 0.86	0.95 (0.33; 2.76) *p* = 0.93
*Guideline combination*						
MVPA + SB						
Met guideline	Reference	Reference	Reference	Reference	Reference	Reference
Did not meet	1.61 (0.49; 5.33) *p* = 0.43	1.78 (0.52; 6.08) *p* = 0.36	1.99 (0.57; 7.00) *p* = 0.28	1.40 (0.44; 4.45) *p* = 0.57	1.15 (0.33; 3.97) *p* = 0.82	1.18 (0.34; 4.09) *p* = 0.80
MVPA+Sleep						
Met guideline	Reference	Reference	Reference	Reference	Reference	Reference
Did not meet	0.69 (0.18; 2.65) *p* = 0.59	0.65 (0.17; 2.53) *p* = 0.53	0.54 (0.13; 2.21) *p* = 0.39	0.53 (0.14; 2.07) *p* = 0.36	0.61 (0.15; 2.56) *p* = 0.50	0.89 (0.19; 4.08) *p* = 0.88
Sleep + SB						
Met guideline	Reference	Reference	Reference	Reference	Reference	Reference
Did not meet	0.86 (0.30; 2.43) *p* = 0.77	0.86 (0.29; 2.52) *p* = 0.78	0.84 (0.29; 2.48) *p* = 0.75	0.62 (0.22; 1.76) *p* = 0.37	0.60 (0.19; 1.87) *p* = 0.37	0.57 (0.18; 1.81) *p* = 0.34
MVPA + SB+Sleep						
Met guideline	Reference	Reference		Reference	Reference	
Did not meet	0.70 (0.16; 3.07) *p* = 0.64	0.93 (0.32; 2.76) *p* = 0.90	‐	0.48 (0.11; 2.18) *p* = 0.34	0.48 (0.10; 2.38) *p* = 0.37	‐
*Guideline clustering*						
Three guidelines met	Reference	Reference		Reference	Reference	
Two guidelines met	0.50 (0.10; 2.62) *p* = 0.41	0.45 (0.08; 2.43) *p* = 0.35	‐	0.45 (0.09; 2.40) *p* = 0.35	0.51 (0.09; 3.08) *p* = 0.46	‐
One guideline met	1.14 (0.24; 5.44) *p* = 0.87	1.11 (0.23; 5.37) *p* = 0.89	‐	0.53 (0.11; 2.60) *p* = 0.43	0.54 (0.10; 2.95) *p* = 0.47	‐
No guideline met	0.33 (0.05; 2.24) *p* = 0.26	0.36 (0.05; 2.44) *p* = 0.29	‐	0.43 (0.07; 2.68) *p* = 0.36	0.33 (0.05; 2.25) *p* = 0.25	‐

a= Considering musculoskeletal disorders at neck, upper back or low back. Model 1= crude; Model 2= adjusted by sex, age and body mass index Model 3= adjusted by variables from Model 2 and for meeting the other guidelines (this model was not supported when considered meeting all guidelines as reference category).

Abbreviations: CI, Confidence Interval; MVPA, Moderate to vigorous physical activity; OR, Odds ratio; SB, Sedentary behavior.

The association of meeting 24‐h movement behavior guidelines with MSD in the low back and spine regions was analyzed by logistic regression and is presented in Table 4. Participants who did not meet both MVPA and sedentary behavior guidelines were more than three times as likely to have MSD in low back (Odds ratio: 3.45 [95% confidence interval: 1.01; 11.88], *p* = 0.049) and in spine (Odds ratio: 3.78 [95% confidence interval: 1.01; 14.15], *p* = 0.048) regions when compared to participants who met these two guidelines, even after adjustment for age, sex, body mass index and for meeting the sleep guideline. These associations showed a posteriori statistical power of 45.1% and 51.9%, respectively. Those participants who did not meet MVPA guideline were four times more likely to have spinal MSD when compared to those who met MVPA guideline (Odds ratio: 4.15 [95% confidence interval: 1.18; 14.52], *p* = 0.03), with a posteriori statistical power of 61.1%. No other significant association has been observed.

**Table 4:** Association of meeting the 24‐h guideline components during Covid‐19 pandemic with low back and spinal musculoskeletal disorders in undergraduate students (*n* = 71).

	Low back MD			Spinal MD^a^		
	Model 1	Model 2	Model 3	Model 1	Model 2	Model 3
	OR (95%CI)	OR (95%CI)	OR (95%CI)	OR (95%CI)	OR (95%CI)	OR (95%CI)
*Individual guidelines*						
MVPA						
Met guideline	Reference	Reference	Reference	Reference	Reference	Reference
Did not meet	2.83 (0.99; 8.09) *p* = 0.05	2.81 (0.96; 8.20) *p* = 0.06	2.60 (0.88; 7.69) *p* = 0.08	**3.78 (1.15; 12.41) *p*** = **0.03**	**3.87 (1.15; 12.99) *p*** = **0.03**	**4.15 (1.18; 14.52) *p*** = **0.03**
SB						
Met guideline	Reference	Reference	Reference	Reference	Reference	Reference
Did not meet	1.57 (0.59; 4.16) *p* = 0.37	1.88 (0.66; 5.34) *p* = 0.24	1.66 (0.56; 4.91) *p* = 0.36	1.13 (0.35; 3.59) *p* = 0.84	0.94 (0.27; 3.24) *p* = 0.92	0.73 (0.19; 2.77) *p* = 0.64
Sleep						
Met guideline	Reference	Reference	Reference	Reference	Reference	Reference
Did not meet	0.91 (0.35; 2.36) *p* = 0.84	0.84 (0.32; 2.24) *p* = 0.73	0.80 (0.29; 2.23) *p* = 0.67	0.67 (0.21; 2.12) *p* = 0.49	0.67 (0.21; 2.19) *p* = 0.51	0.67 (0.20; 2.31) *p* = 0.53
*Guideline combination*						
MVPA + SB						
Met guideline	Reference	Reference	Reference	Reference	Reference	Reference
Did not meet	2.92 (0.91; 9.43) *p* = 0.07	3.33 (0.98; 11.36) *p* = 0.05	**3.45 (1.01;11.88) *p* = 0.049**	3.48 (0.99; 12.22) *p* = 0.05	3.54 (0.97; 12.90) *p* = 0.06	**3.78 (1.01; 14.15) *p*** = **0.048**
MVPA+Sleep						
Met guideline	Reference	Reference	Reference	Reference	Reference	Reference
Did not meet	2.66 (0.68; 10.45) *p* = 0.16	2.59 (0.64; 10.43) *p* = 0.18	2.25 (0.54; 9.36) *p* = 0.27	1.75 (0.39; 7.79) *p* = 0.46	1.86 (0.41; 8.48) *p* = 0.42	1.95 (0.41; 9.34) *p* = 0.40
Sleep + SB						
Met guideline	Reference	Reference	Reference	Reference	Reference	Reference
Did not meet	2.44 (0.85; 7.03) *p* = 0.10	2.37 (0.79; 7.10) *p* = 0.12	2.30 (0.75; 7.05) *p* = 0.15	2.00 (0.60; 6.62) *p* = 0.26	1.96 (0.57; 6.76) *p* = 0.29	1.83 (0.51; 6.62) *p* = 0.35
MVPA + SB+Sleep						
Met guideline	Reference	Reference		Reference	Reference	
Did not meet	2.90 (0.63; 13.26) *p* = 0.17	3.02 (0.64; 14.16) *p* = 0.16	‐	1.28 (0.23; 7.11) *p* = 0.78	1.34 (0.24; 7.60) *p* = 0.74	‐
*Guideline clustering*						
Three guidelines met	Reference	Reference		Reference	Reference	
Two guidelines met	1.83 (0.35; 9.72) *p* = 0.48	1.89 (0.34; 10.45) *p* = 0.47	‐	0.67 (0.11; 4.20) *p* = 0.67	0.74 (0.11; 4.83) *p* = 0.76	‐
One guideline met	4.58 (0.89; 23.73) *p* = 0.07	4.65 (0.88; 24.67) *p* = 0.07	‐	3.00 (0.41; 22.08) *p* = 0.28	3.20 (0.43; 24.08) *p* = 0.26	‐
No guideline met	2.33 (0.37; 14.61) *p* = 0.36	2.50 (0.39; 16.14) *p* = 0.34	‐	1.00 (0.13; 7.89) *p* > 0.99	0.90 (0.11; 7.27) *p* = 0.92	‐

Abbreviations: CI, Confidence Interval; MVPA, Moderate to vigorous physical activity; OR, Odds ratio; SB, Sedentary behavior.

^a^ Considering musculoskeletal disorders at neck, upper back or low back. OR: Odds ratio; CI: Confidence Interval; MVPA: Moderate to vigorous physical activity.; SB: Sedentary behavior. Model 1= crude; Model 2= adjusted by sex, age and body mass index Model 3= adjusted by variables from Model 2 and for meeting the other guidelines (this model was not supported when considered meeting all guidelines as reference category.

## 4 DISCUSSION

The present study analyzed the association between 24‐h movement behaviors with MSD in a sample of undergraduate students during Covid‐19 pandemic. Participants who met sedentary behavior guidelines were more likely to report upper back MSD, while those who met both MVPA and sedentary behavior guidelines were less likely to have low back and spinal MSD than those who did not meet. The MVPA was the only movement behavior which were different according to the presence of spinal MSD, where participants with MSD showed lower MVPA level than those without MSD during Covid‐19 pandemic.

The prevalence of meeting 24‐h movement guidelines was low in this study sample (11.3%). A low prevalence was also observed in a recent large study, where only 1.6% of Latin American adults met the three movement guidelines recommendation.^20^ In this sense, no association was observed between fully meeting the 24‐h movement guidelines with MSD at spine region during Covid‐19 pandemic. Upon this low prevalence, the studies have been considered the analysis of meeting the guidelines individually or in specific combination, which has been positively associated with physical and mental health ^21^ in undergraduate students during Covid‐19 pandemic period. Being healthier as possible was an important self‐action during pandemic, since proportion of undergraduate students who meet physical activity and SB guideline substantially decreased.^22^

Participants with MSD showed lower weekly minutes of MVPA in the present study when compared to those without MSD. In addition, those participants who met MVPA guideline (≥150 min per week) presented lower prevalence of MSD in low back and spine. The pain has been reported as the main barrier for physical activity engagement.^23^ However, the practice of physical activity is recommended even in the presence of MSD, due to several positive factors, such as reduction in the pain severity, improvement in physical functioning and in overall physical and mental health.^24^ However, logistic regression models did not reveal a significant association of meeting MVPA guideline alone with MSD in the present study. Because the physical activity measured by accelerometer is not able to classify into life contexts, it is possible that the independent association of MVPA with MSD could have been mitigated through different domains of physical activity, since occupational physical activity was associated with higher MSD while those at leisure‐time showed an inverse association with MSD.^25^, ^26^

Undergraduate students who met both MVPA and SB guidelines in the present study were less likely to report MSD in low back and in spine region than those who did not meet these guidelines. Previous studies reported that spine region was the most affected by MSD in undergraduate students ^14^, ^27^, ^28^ reported that lifetime prevalence of low back pain is higher and is associated with people insufficiently active and sedentary.^29^ emphasizes that the lower back region is prone to a greater occurrence of disorders that are related to physical inactivity, affecting functionality. In this sense, participants of the present study who did not meet physical activity neither SB guidelines correspond to those with very low levels of daily movements, suggesting that association between physical activity and SB may result in a cumulative effect regarding MSD, which was not achieved individually.

Otherwise, the present study observed that participants who did not meet SB guideline were less likely to have upper back MSD than those who met this guideline. This was a surprising result, but may have a possible hypothesis to support it. It has been reported that people with middle‐to‐high socioeconomic level were less likely to meet SB guideline.^20^ In this sense, people with lower education tends to have higher physical demand in their occupational tasks, resulting in less SB and more chance of meeting SB guideline, but also being more susceptible to have higher spinal MSD,^30^ mainly when compared to sedentary occupational activities.^26^ Thus, the SB may be a complementary proxy of daily activity profile in the association with MSD and needs further investigation, especially regarding different domains of SB, such as occupational and leisure‐time.

Even being reported a bidirectional association between sleep and body pain,^31^ no association was observed between sleep duration and MSD in the present study.^6^ reported a U‐shaped relationship between sleep duration and MSD, where participants with 7 h of sleep time associated with lower MSD when compared with participants with sleep time of ≤5 h or ≥9 h. A poor sleep quality has been associated with an increase in cytokines that can lead to hyperalgesia or increased pain sensitivity. However, this study did not investigate the association of sleep quality indicators with MSD besides sleep duration. A large breadth of evidence supported that a better sleep quality is associated with lower MSD,^32^, ^33^, ^34^, ^35^ but more investigations are needed to propose the integration of sleep quality and duration indicators in 24‐h movement guidelines, mainly when using questionnaires. In addition, it has been observed that pandemic period was associated with higher occurrence of sleep disorders than the periods pre and post pandemic, as well as higher levels of depression than pre‐pandemic period among first‐year university students,^36^ which may have substantially affected the sleep patterns in this population. It is crucial to caveat that pandemic period per se had substantial contribution to harm lifestyle and musculoskeletal health in college students, marked by a sudden isolation with physical and mental consequences, such as mood alterations, substance abuse, lack of social support, health care uncertainty, triggering stressor pathways and increasing central sensitization, which may have led to an impairment of chronic pain.^37^

This study has important limitations, such as the non‐randomization of the sample, the lack of control over the intake of analgesics and the restricted sample, which precluded extrapolation of findings to general student population and limited statistical power of association. Furthermore, this cross‐sectional investigation was not able to infer about pre‐existing MSD in the sample before 12‐month period recalled by Nordic questionnaire, being unable to investigate their influence in the findings. However, as strengths, this study was conducted during a critical period of Covid‐19 pandemic in a highly affected population, physically and mentally. The biosafety procedures allowed to objectively assess the physical activity of sample by accelerometer, which is considered as an important tool for investigation of physical activity patterns among people with MSD.^38^

### 4.1 Implications of physiotherapy practice

From a clinical point of view for health promotion and rehabilitation, the results provide important data for creating interventions to promote increased physical activity associated with a decrease in time spent in sedentary behavior and improved sleep quality, as a strategy protective against musculoskeletal pain, especially in the spine. In this sense, physiotherapeutic actions in the health care network must promote healthy lifestyle habits, which need to focus on the entire 24‐h period (sleep, sedentary behavior and physical activity) instead of prioritizing individual behaviors. The codependency of behaviors shows that the balance between them is associated with health. This therapeutic strategy can be incorporated by Physiotherapists who work at all levels of health care, from primary to specialized care, with adherence to home programs, as professionals provide health guidance related to the intervention to patients. Moreover, post‐pandemic interventions among university students are needed to redeem and promote 24‐h movement guidelines adherence aiming to mitigate MSD and college absenteeism, as well as to improve biopsychosocial health in university students, since previous intervention with multicomponent exercise program in post‐COVID‐19 patients reported to reduce absenteeism and productivity gains.^39^

## 5 CONCLUSION

This study observed that meeting 24‐h movement guidelines was differently associated with MSD in the spine region among undergraduate students during Covid‐19 pandemic. Meeting MVPA and SB guidelines was associated with lower MSD in the low back and in the spine region, while meeting SB guideline alone was associated with higher MSD in the upper back. To the best of our knowledge, this is the first study which investigated the association of meeting 24‐h movement guidelines with MSD in spine region among undergraduate students during Covid‐19 pandemic and these findings may be useful to guide further investigations about lifestyle behaviors and musculoskeletal health in undergraduate students from the pandemic period forward.

## AUTHOR CONTRIBUTIONS

**Gracielle de Jesus Santos:** Conceptualization; methodology; data curation; writing—original draft. **William Rodrigues Tebar:** Methodology; data curation; statistical analysis; writing—original draft. **Danilo Rodrigues Pereira da Silva:** Methodology; writing—review and editing. **Eduardo da Silva Alves:** Methodology; writing—review and editing; funding acquisition. **Diego Giulliano Destro Christofaro:** Methodology; project administration; writing—review and editing. **David Ohara:** Conceptualization; methodology; supervision; project administration; writing—review and editing.

## CONFLICT OF INTEREST STATEMENT

The authors declare no conflicts of interest.

## ETHICS STATEMENT

This study was performed in accordance with the ethical standards as laid down in the 1964 Declaration of Helsinki and its later amendments, and under ethical approval from the ethics committee at the State University of Santa Cruz, DCS/UESC, Brazil.

Ethics Committee on Ethics in Research with Human Beings of the UESC through opinion number 4.832.080.

## TRANSPARENCY STATEMENT

The lead author Eduardo da Silva Alves affirms that this manuscript is an honest, accurate, and transparent account of the study being reported; that no important aspects of the study have been omitted; and that any discrepancies from the study as planned (and, if relevant, registered) have been explained.

## Data Availability

The data that support the findings of this study are available from the corresponding author upon reasonable request.
